# Effects of medical consortium policy on health services: an interrupted time-series analysis in Sanming, China

**DOI:** 10.3389/fpubh.2024.1322949

**Published:** 2024-01-24

**Authors:** Xinmei Yang, Yang Chen, Chengyue Li, Mo Hao

**Affiliations:** ^1^Research Institute of Health Development Strategies, Fudan University, Shanghai, China; ^2^Collaborative Innovation Center of Social Risks Governance in Health, Fudan University, Shanghai, China; ^3^Department of Health Policy and Management, School of Public Health, Fudan University, Shanghai, China; ^4^Department of Hospital Quality Evaluation and Medical Record Management, the Third People’s Hospital of Chengdu, Chengdu, China

**Keywords:** medical consortium, health services, county hospitals, grassroots medical institutions, interrupted time series analysis

## Abstract

**Objectives:**

China has implemented reforms to enhance the operational efficiency of three-level medical services through medical consortiums (MCs). This study evaluated the impact of MCs reform on health services in Sanming, China.

**Methods:**

An interrupted time-series analysis (ITSA) was conducted to assess the impact of MCs on changes in health service levels and trends across the overall situation of MCs and different institutional types within MCs, including county hospitals and grassroots medical institutions. The evaluation focused on various indicators such as outpatient and emergency visits, inpatients, average length of stay, occupancy rate of hospital beds, and hospital bed turnover times. Monthly data were collected from April 2015 to June 2019 through reports on the Sanming Municipal Health Commission website and the Sanming public hospital management monitoring platform.

**Results:**

After the intervention of MCs reform, a significant increase was observed in the total number of inpatients (β_3_ = 174.28, *p* < 0.05). However, no statistically significant change was observed in the total number of outpatient and emergency visits (β_3_ = 155.82, *p* = 0.91). Additionally, the implementation of MCs reform led to an amplification in service volumes provided by county hospitals, with significant increases in the number of outpatient and emergency visits (β_3_ = 1376.54, *p* < 0.05) and an upward trend in the number of inpatients (β_3_ = 98.87, *p* < 0.01). However, no significant changes were observed under the MCs policy for grassroots medical institutions regarding the number of outpatient and emergency visits (β_3_ = −1220.72, *p* = 0.22) and number of inpatients (β_3_ = 75.42, *p* = 0.09).

**Conclusion:**

The Sanming MCs reform has achieved some progress in augmenting service volumes. Nevertheless, it has not led to an increase in service volumes at the grassroots medical institutions. There persists an insufficiency in the efficiency of services and a need for further improvement in primary healthcare. To address these concerns, it is imperative for county hospitals to offer targeted assistance that can enhance motivation among grassroots medical institutions. Besides the MCs should explore initiatives, including improved management of medical equipment, allocation of funding, and personnel resources.

## 1 Introduction

Given the rapidly advancing age demographics among its population, rising burden of chronic disease, increasing prevalence of risk factors, and changing environmental conditions, efficiently allocating health resources for improved patient outcomes has become a policy priority and challenge within the realm of health (1, 2). China has undertaken numerous substantial health policies and initiatives to establish a robust functioning healthcare service infrastructure over recent decades. However, despite incremental enhancements stemming from ongoing comprehensive reforms within China’s healthcare system, scholars’ analysis revealed that approximately 82% of available resources remain concentrated in tertiary and secondary hospitals, whereas merely 18% is allocated toward primary care facilities (3). Consequently, China’s current healthcare system framework grapples with unsustainable predicaments, such as the insufficient distribution of medical resources. Therefore, addressing these concerns becomes an urgent necessity.

As the cornerstone of China’s integrated medical delivery system, the primary objective of medical consortiums (MCs) is to optimize health resource allocation and bolster the capabilities of primary medical institutions. The Chinese government has implemented a series of policies to enhance the efficiency of China’s healthcare system, particularly focusing on MCs. In April 2017, the General Office of the State Council issued “Guidelines on Promoting the Construction and Development of Medical Consortia” (4). Subsequently, in July 2020, the National Health Commission and National Administration of Traditional Chinese Medicine released a policy titled “The Notice on Releasing Management Measures for a Medical Consortium (trial)” (5). Several provinces and cities in China, including Hainan (6), Zhejiang (7), and Guangdong (8), have explored various reforms related to MCs. As of 2020, 4,028 MCs had been established in China (9). However, the formation of MCs is still at an early stage and faces several challenges, such as a lack of clarity regarding institutions and responsibilities and limited primary care capacity (10, 11).

In 2012, the city of Sanming in Fujian Province embarked on a comprehensive healthcare system reform, and has since become a leading healthcare reform model in China. In 2021, the National Health Commission issued the “Notice on the Promotion of Experience in the Construction of Hierarchical Diagnosis and Treatment and Medical Consortium in Sanming,” which summarized the experience of developing compact MCs in Sanming and provided many practical insights into how this could be accomplished. In 2017, Sanming implemented a reform of MCs that relied on county hospitals to form the foundation. This reform oversaw several grassroots medical institutions, including township hospitals and community health centers, to improve regional resource allocation and optimize medical resource utilization. The objectives of Sanming’s MCs policy can be summarized in two aspects: First, to promote the equalization of health resources and hierarchical diagnosis and treatment, as well as resolve issues with unreasonable medical treatment orders, to provide health services “at their doorsteps” for the public and establish an orderly system for medical care. Second, to encourage county hospitals to take the lead in implementing precise management policies and expanding technology and services at the grassroots level, thereby comprehensively enhancing the overall capacities of grassroots medical institutions. In order to achieve the above objectives, the following major measures have been undertaken. First, county hospitals within the compact MCs are responsible for resource allocation. Second, MCs should standardize internal management, including human resources management, funding and asset allocation, performance measurement and information construction. Third, payment reform related to the medical insurance package should create an economic incentive to retain a balance in the medical insurance fund. To date, MCs have been formed in all counties in Sanming, involving 18 county-level hospitals and 144 township hospitals and community health centers, making 10 compact county-level MCs. This would ultimately improve the service capabilities and operational efficiency of county hospitals and grassroots medical institutions in MCs. Therefore, paying more attention to the effects before and after implementing the MCs policy is important.

Several studies have previously reported on the status of China’s MCs reform (8), regional practical experiences (12), theoretical frameworks (13), and development pathways (14). Although some studies have examined the effects of MCs policy using quantitative analyses, most of them have been conducted from a single perspective, such as assessing inpatient numbers (15), hospitalization rates (16), hospitalization expenses (17), physician flows (18), informatization construction (19), and health professionals’ views and satisfaction with regard to MCs reform (20). For example, Özkaytan et al. (21) found that integrated care models could address complicated healthcare needs and enhance service continuity and coordination. Ye et al. (22) used the super-efficiency slack-based measure–data envelopment analysis (SBM-DEA) model to demonstrate that MCs reform could improve the efficiency of medical services provided by county-level public general hospitals. Similarly, using the standardized patient approach, Su and Zhou (23) found that the MCs model could improve the quality of primary medical services. After the MCs reform, Yuan et al. (24) found that county-level public hospitals improved their service efficiency and capability.

Many scholars also researched the effects of the Sanming Model after its comprehensive healthcare system reform (25). For instance, Fu et al. focused on medical costs (26), Meng et al. analyzed drug expenses (27), and Hu et al. paid attention to the use of antibiotics (28), which confirmed that the Sanming medical model has positive effects. Researchers have paid attention to Sanming’s MCs reforms as a research topic and analyzed the current situation (29), governance mechanism (9), and practical experience (30). However, only a few studies have measured the effects of Sanming MCs reform. Liao et al. conducted a descriptive analysis to report that the MCs model helped optimize the hospital income structure (31). Li et al. used a stepwise regression model to demonstrate that the MCs reform positively affected the structure of medical expenses (32). Some studies have examined the effects of MCs reform in a particular county. For example, Zheng et al. interviewed 15 key informants and found that establishing compact MC in Youxi County helped prompt professional integration (33). Zhong ZD et al. used an interrupted time-series analysis (ITSA) to report that the number of surgeries had decreased in county hospitals (34). Zhong ZC et al. employed an ITSA to analyze that the cost of outpatient and emergency patients per time in Youxi County hospitals increased after the medical insurance payment reform (35). Xiong et al. researched the overall, grassroots, and hospitals, which used an ITSA to find that economic incentives of MCs policy have influenced the number of inpatient, outpatient and emergency visits (36). However, they mostly focused on the overall effects or a specific medical institution. Therefore, quantitative research focusing on evaluating the observable effects on health services after the MCs policy implementation, considering both the overall situation of MCs and the different types of institutions within MCs, could provide a more accurate picture of the overall impact of MCs reform.

This study aimed to analyze the effects of Sanming’s MCs reform on health services as judged by the overall situation of MCs and different institutional types (county hospitals and grassroots medical institutions) within MCs to understand the problems and limitations of China’s MCs policy. The findings from this research will serve as a valuable reference for MCs reform in China and other developing countries, facilitating the comprehensive and effective implementation of an integrated health service system.

## 2 Materials and methods

### 2.1 Study setting and design

Sanming is a prefecture-level city in Fujian province, with a residential population of 2.455 million, and a gross domestic product (GDP) *per capita* of ¥126,044 in 2022. As of 2022, 7,463 registered physicians/physician assistants, 9,118 registered nurses, and 16,843 hospital beds were available in Sanming (37).

In April 2017, the Sanming Municipal Committee and Municipal Government issued the “Opinions on Implementation of General Hospitals in the Establishment,” aiming to establish high-level general hospitals in the region. This helped select the intervention point for evaluating the effects of the MCs policy in the present study. Considering the time required for the reforms to be implemented, May 2017 was set as the time boundary, as the county hospitals and grassroots medical institutions needed to prepare for the implementation of MCs, including establishment of organizational structures, division of responsibilities and operational mechanisms. April 2015 was selected as the initial date of pre-MCs adoption, and June 2019 was the end date of post-MCs adoption, ensuring equal time (25 months) before and after the adoption of the MCs reforms to facilitate a meaningful comparison (38). We used an ITSA to assess the impact of the MCs policy in Sanming on trend changes in health services provision from April 2015 to June 2019. It ensured that this study had enough data points to assess the intervention’s effect (39).

### 2.2 Indicator evaluation and measurement

Based on the policy objectives proposed by the Sanming MCs reform, this study aimed to assess the effects of constructing MCs from two aspects. One focused on the fact that the construction of MCs should serve as an integration mechanism between different health institutions in the county, helping to form an orderly system for medical care. Therefore, this study used two indicators, the total number of outpatient and emergency visits and the total number of inpatients, to focus on intra-county integration based on the overall situation of county and township medical institutions in the region (40). The other was to improve the health service efficiency of county hospitals and grassroots medical institutions under the MCs. Therefore, this study also analyzed the changes in service indicators for county hospitals and grassroots medical institutions separately. The analysis and evaluation of outpatient and inpatient services were representative for assessments of the hospitals’ efficiency and management levels. In summary, indicators such as the number of outpatient and emergency visits, number of inpatients, average length of stay, occupancy rate of hospital beds, and hospital bed turnover times were utilized (41, 42). The relevant formulas used are as follows:


$$\mathrm{Average}\;\mathrm{length}\;\mathrm{of}\;\mathrm{stay}=\frac{\mathrm{Occupied}\;\mathrm{bed}-\mathrm{days}\;}{\mathrm{Number}\;\mathrm{of}\;\mathrm{discharged}\;\mathrm{patients}}$$

$$\mathrm{Occupancy}\;\mathrm{rate}\;\mathrm{of}\;\mathrm{hospital}\;\mathrm{beds}=\frac{\mathrm{Occupied}\;\mathrm{bed}-\mathrm{days}\;}{\mathrm{Active}\;\mathrm{bed}-\mathrm{days}}$$

$$\mathrm{Hospital}\;\mathrm{bed}\;\mathrm{turnover}\;\mathrm{times}=\frac{\mathrm{Number}\;\mathrm{of}\;\mathrm{discharged}\;\mathrm{patients}\;}{\mathrm{Average}\;\mathrm{of}\;\mathrm{active}\;\mathrm{beds}}$$

The indicators used in this study are routinely collected by the medical statistics department in county public hospitals and grassroots medical institutions and reported to the Municipal Health Commission in Sanming. It was assumed that the trend changes in these indicators resulted from Sanming’s implementation of MCs.

### 2.3 Data sources

Monthly data were collected from 10 county MCs, including 18 county-level public hospitals and 144 grassroots medical institutions from April 2015 to June 2019. The post-policy period was 25 months from June 2017 to June 2019, and the baseline data period was 25 months, covering the period from April 2015 to April 2017. All indicators were collected from monthly reports on the Sanming Municipal Health Commission website and the Sanming public hospital management monitoring platform.

### 2.4 Data analysis

ITSA is commonly used to analyze the effects of interventions in situations where a control group is difficult or impossible to find, and the period for data collection can be divided into two time segments (pre- and post-intervention) to be analyzed using a regression model (38, 39, 43). Because no control group was available, we used an ITSA to evaluate the impact of the MCs policy intervention. Accordingly, we constructed the following model:


$$\begin{array}{l} Y=\beta_{0}+\beta_{1}\times \mathrm{Time}+\beta_{2}\times \mathrm{Intervention}\\ \;+\beta_{3}\times \mathrm{Time}\;\mathrm{after}\;\mathrm{Intervention}+\beta_{i}\times C+\varepsilon_{t} \end{array}$$

where *Y* is the dependent variable representing the number of outpatient and emergency visits, number of inpatients, average length of stay, occupancy rate of hospital beds, and hospital bed turnover times. The independent variables included time, intervention, and time after intervention. “Time” refers to each month from April 2015 to June 2019. “Intervention” is a binary variable coded as 0 for the period before and 1 for the period after the intervention. “Time after intervention” equals 0 before policy implementation and increases monthly afterward. Moreover, GDP per quarter and quarterly dummy variables were included as control variables. C refers to the control variable. As we only collected annual GDP data from 2015 to 2016 and quarterly GDP data from 2017 to 2019, we extrapolated the quarterly GDP from 2015 to 2016 based on the quarterly GDP composition ratios from 2017 to 2019. β_0_, β_1_, β_2_, and β_3_ reflect the baseline level, time trend without considering the intervention, immediate effects of the intervention on the level of selected indicators, and continuous effect of the intervention on the trend of indicators, respectively. ε is the error term.

In this study, ITSA was performed with Newey-West standard errors, and all indicators were investigated for autocorrelations using the Cumby-Huizinga test (44). If autocorrelation was present at higher lag orders (up to the six lags tested), the lag orders accounted for it. All analyses were performed using Stata version 15 (StataCorp., College Station, TX, United States), and a *p*-value < 0.05 was considered statistically significant.

## 3 Results

### 3.1 Description analysis of health services

Table 1 presents the descriptive statistics. The mean total number of outpatient and emergency visits and the mean total number of inpatients increased after the reforms. In county hospitals, the mean number of outpatient and emergency visits, the number of inpatients, the average length of stay, and the occupancy rate of hospital beds increased. However, during the policy’s implementation, the mean hospital bed turnover times gradually decreased from 3.16 to 2.95. Correspondingly, the mean number of outpatient and emergency visits for grassroots medical institutions increased from 232135.08 to 356592.04. The mean number of inpatients, average length of stay, occupancy rate of hospital beds, and hospital bed turnover time were 8739.80, 5.19, 47.30, and 2.79, before the reform and 8498.20, 5.17, 46.87, and 2.75 afterward, respectively.

**Table 1:** Descriptive statistics of health services indicators.

Indicators	Mean (SD)	
	Pre-intervention	Post-intervention
Total number of outpatient and emergency visits (n)	534128.24 (58399.77)	737112.76 (67484.22)
Total number of inpatients (n)	24994.20 (2148.03)	26746.24 (2694.98)
**County hospitals**		
Number of outpatient and emergency visits (n)	301993.16 (20786.06)	380520.72 (36669.00)
Number of inpatients (n)	16254.40 (746.96)	18248.04 (1695.75)
Average length of stay (days)	7.87 (0.49)	8.60 (0.65)
Occupancy rate of hospital beds (%)	83.89 (4.74)	85.59 (4.36)
Hospital bed turnover times (n)	3.16 (0.20)	2.95 (0.22)
**Grassroots medical institutions**		
Number of outpatient and emergency visits (n)	232135.08 (43118.00)	356592.04 (36457.67)
Number of inpatients (n)	8739.80 (1682.73)	8498.20 (1470.47)
Average length of stay (days)	5.19 (0.25)	5.17 (0.22)
Occupancy rate of hospital beds (%)	47.30 (6.22)	46.87 (7.72)
Hospital bed turnover times (n)	2.79 (0.50)	2.75 (0.44)

### 3.2 Effects of MCs policy on health services

**Figure 1:**
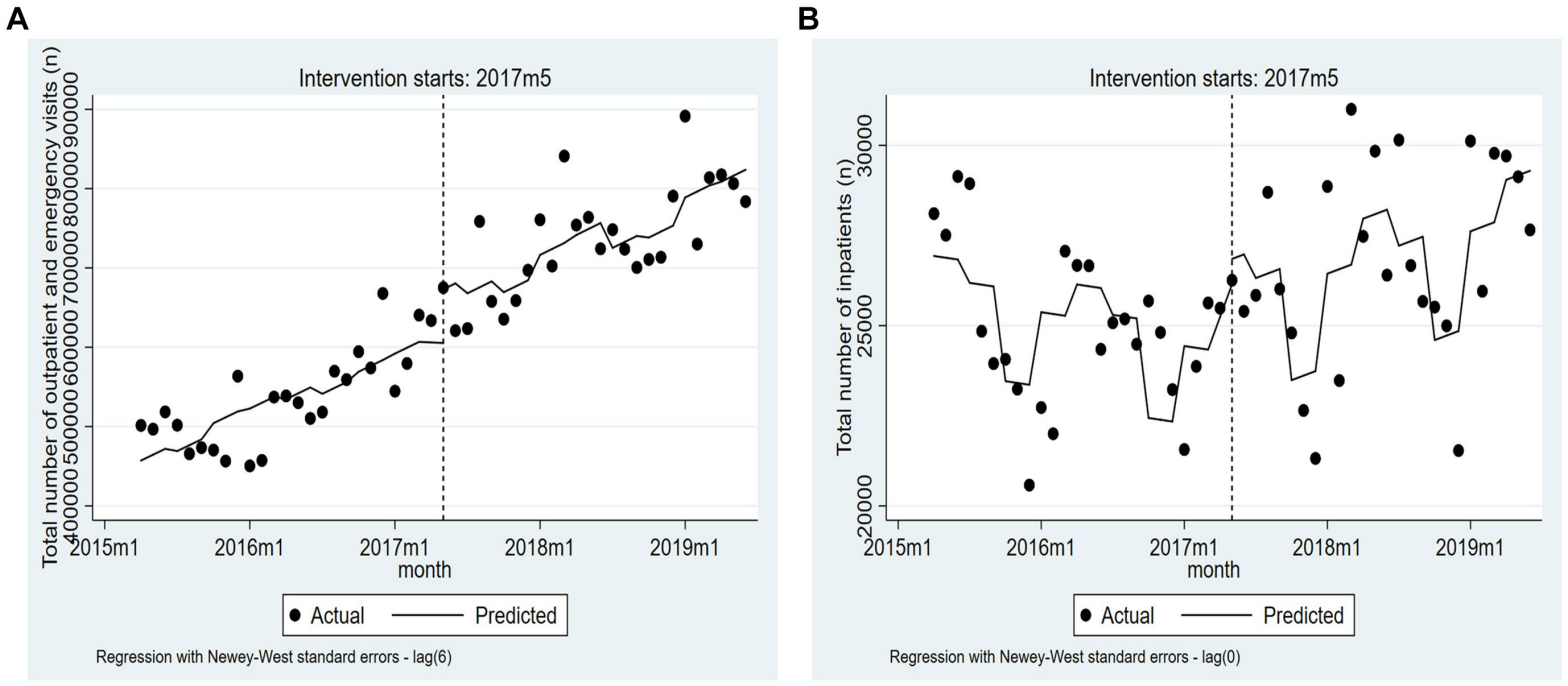
The trend changes of two indicators based on the adjusted model (from April 2015 to June 2019). **(A)** Total number of outpatient and emergency visits. **(B)** Total number of inpatients.

All evaluation results of ITSA are presented in Table 2. Model 1 is a crude model. Model 2 is the adjusted model, in which we control for quarterly GDP and quarterly dummy variables. Table 2 and Figure 1 indicate that, after the policy intervention, the trend change of the total number of inpatients significantly increased (β_3_ = 174.28, 95% confidence interval [CI] = 37.62–310.94, *p* < 0.05). However, no significant changes were observed in the total number of outpatient and emergency visits for the trend (β_3_ = 155.82, 95% CI = −2536.03 to 2847.66, *p* = 0.91).

**Table 2:** Evaluation results of interrupted time-series analysis for the policy effects on the health services indicators in county and grassroots medical institutions.

Indicators	Baseline level, β_0_ (95% CI)	Baseline trend, β_1_ (95% CI)	Level change, β_2_ (95% CI)	Trend change, β_3_ (95% CI)
**Total number of outpatient and emergency visits (n)**				
Model 1	459642.90^***^ (429350.60, 489935.20)	6207.11^***^ (4045.86, 8368.36)	43186.44 (−6051.66, 92424.54)	−69.32 (−3320.82, 3182.17)
Model 2	623436.40^***^ (402059.80, 844813.10)	7358.83^**^ (3149.62, 11568.04)	59598.30^*^ (10277.95, 108918.70)	155.82 (−2536.03, 2847.66)
**Total number of inpatients (n)**				
Model 1	26216.45^***^ (23538.11, 28894.78)	−101.85 (−253.70, 50.00)	1794.87 (−576.72, 4166.45)	202.86^*^ (7.96, 397.76)
Model 2	28880.00^***^ (18493.71, 39266.29)	−49.27 (−185.02, 86.47)	1658.73 (−603.59, 3921.06)	174.28^*^ (37.62, 310.94)

Model 1: Crude model;Model 2: Adjusted for quarterly gross domestic product, and quarterly dummy variables.

*p < 0.05.

**p < 0.01.

***p < 0.001.

**Figure 2:**
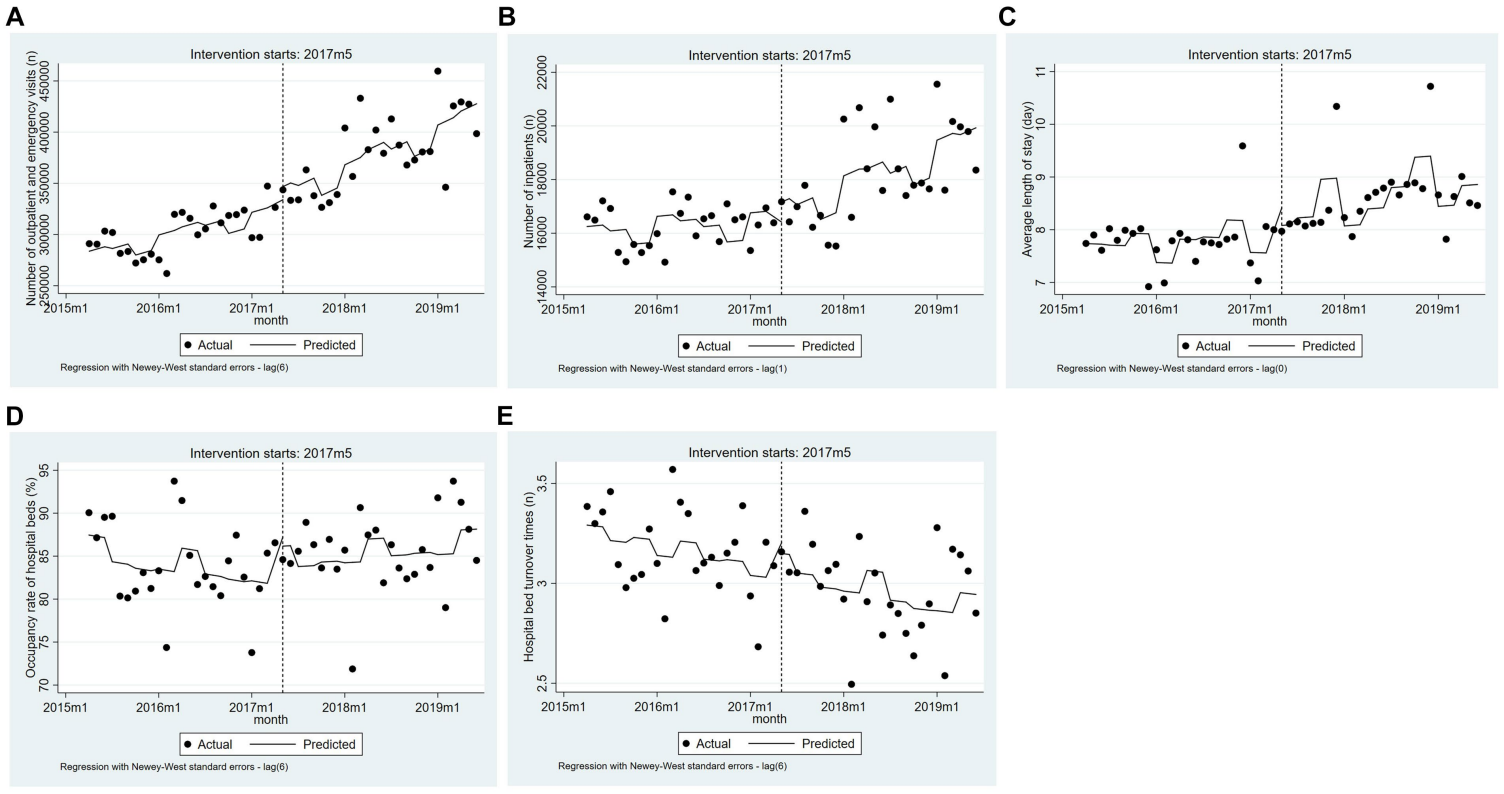
The trend changes of five indicators in county hospitals based on the adjusted model (from April 2015 to June 2019). **(A)** Number of outpatient and emergency visits. **(B)** Number of inpatients. **(C)** Average length of stay. **(D)** Occupancy rate of hospital beds. **(E)** Hospital bed turnover times.

Table 3 and Figure 2 show the effects of the MCs policy implementation on health service indicators in county hospitals. The baseline trend in outpatient and emergency visits before the policy intervention showed a significant increase (β_1_ = 2179.84, 95% CI = 965.87–3393.82, *p* < 0.01). Meanwhile, the number of outpatient and emergency visits (β_3_ = 1376.54, 95% CI = 70.88–2682.19, *p* < 0.05) showed an increasing trend after the policy intervention. Additionally, the number of inpatients also showed an increasing trend, with a statistically significant β_3_ of 98.87 (95% CI = 32.68–165.05, *p* < 0.01). No significant differences were observed in the trend change for average length of stay, occupancy rate of hospital beds, or hospital bed turnover times.

**Table 3:** Evaluation results of the interrupted time-series analysis for the policy effects on the health services indicators of county hospitals.

Indicators	Baseline level, β_0_ (95% CI)	Baseline trend, β_1_ (95% CI)	Level change, β_2_ (95% CI)	Trend change, β_3_ (95% CI)
**Number of outpatient and emergency visits (n)**				
Model 1	280373.90^***^ (263704.30, 297043.60)	1801.60^***^ (903.46, 2699.75)	12648.57 (−5533.93, 30831.07)	1481.61^*^ (278.57, 2684.66)
Model 2	316017.90^***^ (225195.80, 406840.00)	2179.84^**^ (965.87, 3393.82)	14581.58 (−6643.78, 35806.94)	1376.54^*^ (70.88, 2682.19)
**Number of inpatients (n)**				
Model 1	16071.08^***^ (15432.27, 16709.88)	15.28 (−22.94, 53.50)	290.98 (−754.48, 1336.45)	101.74^**^ (31.00, 172.48)
Model 2	18126.26^***^ (12833.87, 23418.64)	27.31 (−39.92, 94.54)	519.32 (−459.63, 1498.28)	98.87^**^ (32.68, 165.05)
**Average length of stay (days)**				
Model 1	7.69^***^ (7.42, 7.96)	0.01 (−0.02, 0.04)	0.39 (−0.28, 1.06)	0.02 (−0.02, 0.06)
Model 2	5.19^**^ (1.49, 8.88)	−0.01 (−0.05, 0.04)	0.10 (−0.43, 0.63)	0.02 (−0.02, 0.05)
**Occupancy rate of hospital beds (%)**				
Model 1	85.81^***^ (81.65, 89.97)	−0.16 (−0.41, 0.09)	2.42 (−1.27, 6.11)	0.26 (−0.05, 0.56)
Model 2	81.43^***^ (63.40, 99.45)	−0.15 (−0.35, 0.06)	1.79 (−1.09, 4.67)	0.19 (−0.04, 0.43)
**Hospital bed turnover times (n)**				
Model 1	3.27^***^ (3.14, 3.41)	−0.01^*^ (−0.02, −0.002)	0.03 (−0.14, 0.19)	0.001 (−0.01, 0.01)
Model 2	3.60^***^(2.45, 4.74)	−0.004 (−0.01, 0.01)	0.04 (−0.13, 0.20)	−0.0001 (−0.01, 0.01)

Model 1: Crude model;Model 2: Adjusted for quarterly gross domestic product, and quarterly dummy variables.

*p < 0.05.

**p < 0.01.

***p < 0.001.

**Figure 3:**
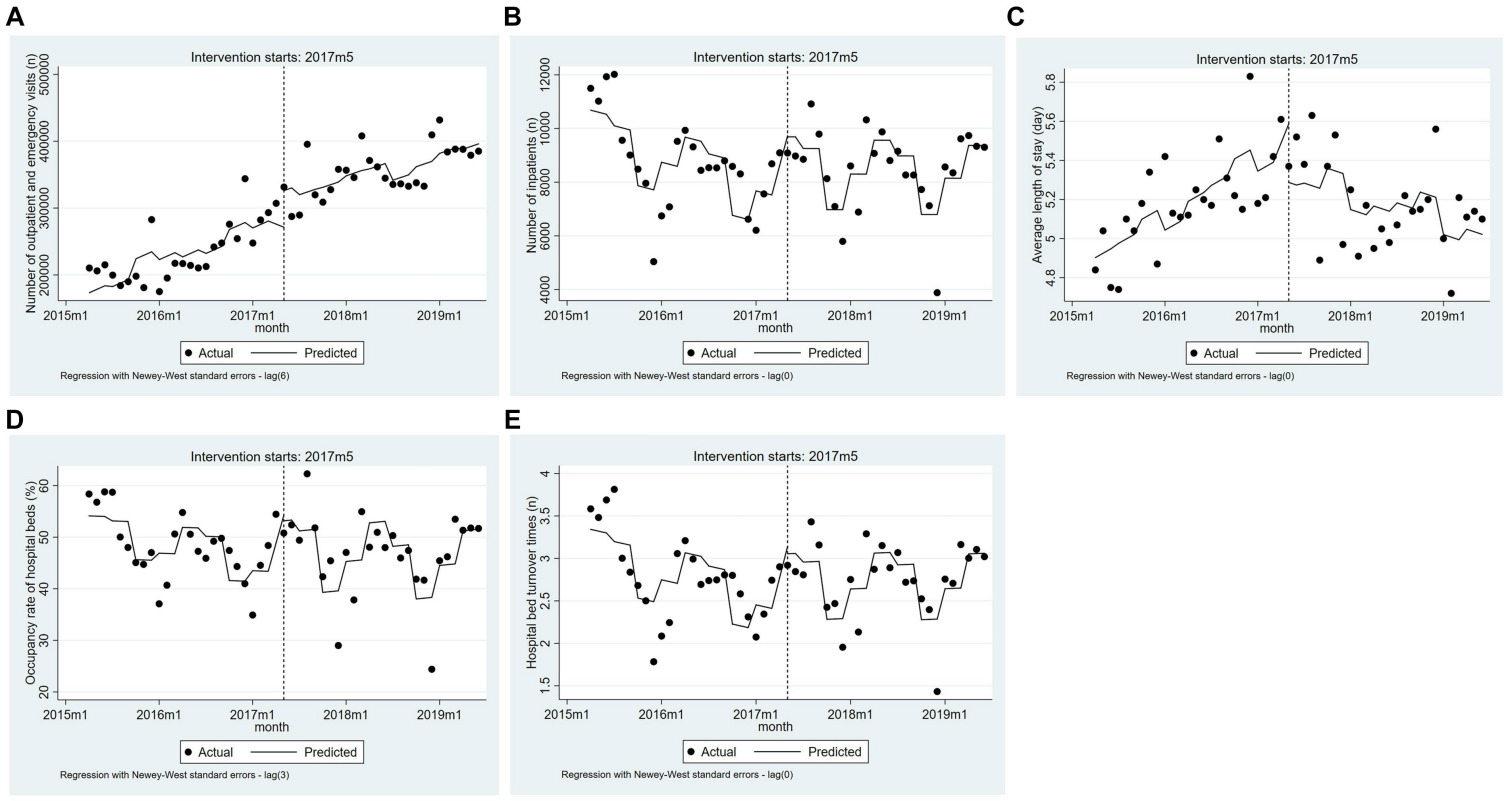
The trend changes of five indicators for grassroots medical institutions based on the adjusted model (from April 2015 to June 2019). **(A)** Number of outpatient and emergency visits. **(B)** Number of inpatients. **(C)** Average length of stay. **(D)** Occupancy rate of hospital beds. **(E)** Hospital bed turnover times.

As Table 4 shows, after the MCs policy implementation, the average length of stay at grassroots medical institutions recorded a statistically significant decrease, with a β_3_ of −0.04 (95% CI = −0.05 to −0.02, *p* < 0.001). Meanwhile, the ITSA did not show significant differences in the trend changes for other indicators (Figure 3).

**Table 4:** Evaluation results of the interrupted time-series analysis for the policy effects on the health services indicators of grassroots medical institutions.

Indicators	Baseline level, β_0_ (95% CI)	Baseline trend, β_1_ (95% CI)	Level change, β_2_ (95% CI)	Trend change, β_3_ (95% CI)
**Number of outpatient and emergency visits (n)**				
Model 1	179269.00^***^ (157512.00, 201026.00)	4405.51^***^ (2827.18, 5983.83)	30537.87 (−4563.41, 65639.14)	−1550.94 (−3752.24, 650.37)
Model 2	307418.50^***^ (151521.00, 463316.10)	5178.99^**^ (2022.53, 8335.45)	45016.72^*^ (9018.93, 81014.52)	−1220.72 (−3178.85, 737.40)
**Number of inpatients (n)**				
Model 1	10145.37^***^ (8039.08, 12251.66)	−117.13^*^ (−232.83, −1.43)	1503.88^*^ (14.25, 2993.52)	101.12 (−51.24, 253.48)
Model 2	10753.74^**^ (3066.24, 18441.25)	−76.58 (−162.82, 9.66)	1139.41 (−181.59, 2460.41)	75.42 (−10.89, 161.73)
**Average length of stay (days)**				
Model 1	4.91^***^(4.77, 5.04)	0.02^***^(0.01, 0.03)	−0.18 (−0.42, 0.07)	−0.04^***^ (−0.05, −0.02)
Model 2	4.55^***^ (3.38, 5.72)	0.02^**^ (0.01, 0.04)	−0.22 (−0.47, 0.03)	−0.04^***^ (−0.05, −0.02)
**Occupancy rate of hospital beds (%)**				
Model 1	53.02^***^ (47.06, 58.97)	−0.39 (−0.81, 0.04)	5.09 (−3.65, 13.83)	0.28 (−0.36, 0.92)
Model 2	71.77^***^ (46.70, 96.83)	−0.05 (−0.46, 0.36)	4.41 (−1.86, 10.68)	0.20 (−0.16, 0.57)
**Hospital bed turnover times (n)**				
Model 1	3.19^**^(2.56, 3.83)	−0.03 (−0.07, 0.001)	0.43 (−0.01, 0.86)	0.03 (−0.01, 0.08)
Model 2	3.33^*^(1.22, 5.45)	−0.02 (−0.05, 0.004)	0.30 (−0.09, 0.69)	0.02 (−0.001, 0.05)

Model 1: Crude model;Model 2: Adjusted for quarterly gross domestic product, and quarterly dummy variables.

*p < 0.05.

**p < 0.01.

***p < 0.001.

## 4 Discussion

Our study focused on Sanming, a prefecture-level city in China. A 51-month data series and ITSA were employed to evaluate the effects of the MCs’ policy implementation on seven health service indicators. This study presented novel empirical evidence regarding the effects of the MCs reform in China and provided scientifically grounded recommendations for other prefecture-level cities aiming to enhance their policy implementation efforts.

This study found no statistically significant change in the total number of outpatient and emergency visits after the MCs reform. However, further analysis revealed a noteworthy increase in such visits at county hospitals while observing no substantial alteration at grassroots medical institutions. These contrasting findings challenged prior research reporting an evident surge in outpatient and emergency visits at grassroots medical institutions after the establishment of MCs. For example, Gong et al. reported that following the implementation of the Luohu Model, the number of patients in grassroots medical institutions increased from 530,000 to 1.99 million people from 2014 to 2019 (45). Similarly, Wang et al. revealed that outpatient and emergency visits at the primary care level increased after implementing the Zhenjiang Model (46). The result of this study suggested inadequate patient retention within these establishments and highlighted deficiencies in establishing appropriate healthcare protocols. There may be several reasons to explain this phenomenon. First, the payment reform of the medical insurance package has created an effective incentive for Samnang’s MCs to improve their health service capabilities and retain a balance in the medical insurance fund. However, county hospitals, leading hospitals with management rights over the fund, have not motivated grassroots medical institutions enough. Second, county hospitals exhibit deficiencies in managing grassroots medical institutions within MCs (47). In Sanming, county hospitals mainly manage grassroots medical institutions by establishing of primary management departments, comprising two to eight employees with diverse professional backgrounds including nursing and pharmacy. However, this approach lacks sufficient professional management talent. Third, county hospitals in Sanming primarily focus on the assessment and fund allocation of grassroots medical institutions instead of prioritizing the long-term development of regional medical institutions. As a result, there is a failure to motivate grassroots medical institutions to enhance their service capabilities adequately. Unlike the Zhenjiang Model, which set up the joint consultation sessions of general practitioners and specialists at the primary. Upper-level hospitals provided management and business cooperation to improve the technical level of grassroots medical institutions (48).

Our findings revealed a decrease in the average length of stay at grassroots medical institutions during the intervention of MCs, aligning with the findings of Lao et al. (49). Generally, the reduction in the average length of stay indicates an optimization in health service allocation and an improvement in the quality of medical services. Consequently, it raises awareness, enhances competitiveness, encourages more patients to seek medical treatment, and promotes sustainable development. This positive outcome may be attributed to the establishment of a unified management system between county hospitals and grassroots medical institutions under the MCs policy implementation. This facilitated the transfer of medical equipment, personnel, technical skills and management expertise from county hospitals to grassroots facilities, enhancing the service capacity of grassroots medical institutions and contributing to the improvement (50). Additionally, the reform in payment for medical insurance packages standardized the diagnosis and treatment process (51). Furthermore, it effectively addressed issues such as excessive treatment for common diseases and unnecessary healthcare services provided by grassroots medical institutions (52).

In the MCs policy implementation context, the total number of inpatients showed a statistically significant increase. The similar finding could also be found in post-MCs reform in Deqing County, Zhejiang (53), and in Youxi County, Sanming (34). From 2017 to 2019, the hospitalization rate for transfer among urban workers’ medical insurance patients in Sanming decreased from 6.84, 6.63% and then to 6.41%. This downward trend may be attributed to the implementation of a medical insurance package, enabling MCs to control transferred inpatients and retain more inpatients within the county hospitals. Furthermore, no significant change was observed in the number of inpatients at grassroots medical institutions, which was consistent with the findings of another study considering the effect of Sanming’s MCs reform (36). It also could be seen that there was a substantial increase in the number of inpatients at county hospitals, consistent with previous studies by Zhao et al. (54) and Tao et al. (55). The findings indicated that county hospitals effectively retained inpatients within their respective counties and exerted control over patients’ outflow. Tertiary hospitals with robust, comprehensive capabilities in provincial and municipal areas could offer valuable support to county hospitals. For example, Fujian Province stipulated that 47 tertiary hospitals at the provincial and municipal level deploy 516 medical personnel to assist county hospitals. This measure aimed to enhance the medical service capacity of county hospitals while promoting local treatment-seeking behavior (56). However, the implementation of MCs policy had little impact on the number of inpatients at grassroots medical institutions. This could be attributed to several reasons. First, a previous study revealed that most incremental inpatient service volumes at grassroots medical institutions were derived from transfers from specialized departments at county hospitals (57). County hospitals heavily relied on inpatient revenue as a significant source of their overall medical revenue. County hospitals in Sanming generated the largest proportion of their total revenue from inpatient services, accounting for 50.61, 49.23, and 46.33%, respectively, in 2017, 2018, and 2019. Community interest consciousness is lacking, resulting in county hospitals’ unwillingness to transfer inpatients to grassroots medical institutions. Consequently, the integration of medical institutions within MCs may further deplete the resources of grassroots medical institutions as higher-level hospitals are reluctant to transfer patients downward. This situation led to inadequate treatment at lower-tiered healthcare centers. Moreover, outpatient and inpatient services have distinct functional roles in disease prevention and the medical service chain. Diseases that require hospitalization pose greater challenges in terms of treatment. However, the limited availability of medical services at grassroots medical institutions led residents to prefer seeking care at higher-level medical institutions with more advanced medical technology and guaranteed quality for hospitalization (58). As a result, their healthcare-seeking behaviors were less significantly affected by the MCs policy (36).

This study also found no statistically significant differences in the average length of stay, occupancy rate of hospital beds, or hospital bed turnover times before and after the MCs reform in county hospitals. This result differed from that in other studies. Ye and Jiang found that, in the long term, the Luohu Model reduced the length of stay in inpatient care (59). These findings indicated that the service capacity of county hospitals has not yet improved, and their leading function has not been adequately emphasized. Therefore, further promotion of county hospitals is necessary to achieve the goal of “county hospitals taking a leading position” in the MCs reform (60). There are still some challenges due to variations in medical care levels, population sizes, economic development between counties, as well as the relatively short period since implementing reform in most county hospitals (61). Given the inadequate infrastructure, weak technology and professionalism, insufficient medical equipment and health personnel, and the lack of interconnected information systems within the county, it was challenging to meet residents’ multilevel and multiple healthcare service demands (62–64). Unlike the Luohu Model, technologies, such as Internet Plus, were utilized to facilitate the sharing of diagnosis and treatment information in the Luohu Medical Group, improving medical services’ efficiency (65). According to the MCs policy, county hospitals play a vital role in ensuring major diseases which can be treated without patients leaving their counties (66). The above findings suggested that county hospitals should define their positioning and expand their diagnosis coverage by strengthening the comprehensiveness of diagnosis and treatment projects. At the same time, improvements are needed in infrastructure, information systems, health personnel, medical equipment, and discipline construction (64).

No significant differences were found in the occupancy rates or turnover times for hospital beds during the MCs policy implementation, indicating a need to strengthen healthcare efficiency in grassroots health institutions (54). These findings may be attributed to several factors, including inadequate health personnel at the grassroots level and uneven distribution of their skills (67). As seen from the MCs monitoring index value of Fujian Province, Sanming had a higher number of daily visits per grassroots doctor at 13.00 compared to the provincial level (11.21). Moreover, some researchers have revealed that Sanming lacks attractive programs to motivate medical personnel to serve at the grassroots level (68). The demanding workload of grassroots health personnel in Sanming increases the likelihood of burnout and turnover among healthcare workers, making recruiting staff for grassroots medical institutions difficult and leading to a personnel shortage and a vicious circle (69). Furthermore, despite receiving technical support from professionals at county-level or tertiary hospitals, the service capacity of grassroots medical institutions remains low. Consequently, professionals in grassroots medical institutions were in subordinate positions (33), thereby limiting the role of primary medical staff and diminishing their motivation to engage actively in the establishment of MCs. Another contributing factor could be patients’ lack of confidence in grassroots hospitals’ quality of care. Patients were not aware of the enhanced convenience offered by grassroots medical institutions for managing non-critical conditions, particularly prevalent chronic diseases (67, 70). Therefore, it is imperative for the governmental health department to formulate human resource policies and measures to augment intrinsic motivation and elevate service standards in grassroots medical institutions. This can be accomplished by implementing incentive policies, bolstering development programs, augmenting remuneration packages, and enhancing working conditions (68). Meanwhile, grassroots medical practitioners must establish robust patient connections by providing guidance and policy advocacy in basic public health services (71), thereby fostering a greater willingness to attend grassroots medical institutions (70).

## 5 Conclusion

Compared to other studies evaluating the effect of the Sanming’s MCs, this study assessed the effects considering the overall situation of MCs and the diverse institutions within them. This comprehensive perspective sheds light on the evaluation of MCs. Our study revealed that implementing the MCs policy in Sanming has partially contributed to the development of healthcare system capabilities. However, it is evident that an appropriate medical order has not yet been established. As key healthcare providers and leaders in the tertiary medical system, county hospitals must strengthen their leading roles further. In addition, grassroots medical institutions have been unable to effectively serve as gatekeepers. This resulted in a siphoning effect whereby patients prefer higher-level hospitals. Addressing this issue is crucial when implementing the MCs policy in other areas. County hospitals and grassroots medical institutions within MCs should formulate long-term development plans for enhancing policy coordination through allocating resources such as medical equipment, funds, and personnel. This approach aims to strengthen health service capacity, improving the service quality and efficiency, reshape patients’ healthcare-seeking habits, and promote comprehensive and continuous medical services in county hospitals and grassroots medical institutions.

### 5.1 Limitations

This study had some limitations. First, the samples used in this study were limited to a single city in eastern China, which restricts the generalizability of the findings. Second, other policies may have influenced the results of ITSA, and its application could not fully mitigate this impact (72). For example, the implementation of diagnosis-related group reform or high-quality related hospital reform in Sanming could have affected health service efficiency. This might have led to an overestimation of the intervention effects of the MCs policy. Third, due to the limitations in data availability, we only collected annual GDP data from 2015 to 2016 and quarterly GDP data from 2017 to 2019 in Sanming. This may influence the results obtained from the adjusted ITSA model. Lastly, evaluating the implementation effect of the MCs policy in Sanming for only approximately 4 years may somewhat compromise the stability of evaluation results. Future research should conduct more in-depth analysis by collecting additional data and comparing it with other Chinese provinces.

## Data availability statement

The raw data supporting the conclusions of this article will be made available by the authors, without undue reservation.

## Author contributions

XY: Data curation, Writing – original draft. YC: Writing – original draft, Data curation. CL: Conceptualization, Supervision, Writing – review & editing. MH: Conceptualization, Supervision, Writing – review & editing.
